# The *Drosophila melanogaster* Levodopa-Induced Depression Model Exhibits Negative Geotaxis Deficits and Differential Gene Expression in Males and Females

**DOI:** 10.3389/fnins.2021.653470

**Published:** 2021-05-17

**Authors:** Thiago C. Moulin, Federico Ferro, Angela Hoyer, Pierre Cheung, Michael J. Williams, Helgi B. Schiöth

**Affiliations:** ^1^Functional Pharmacology Unit, Department of Neuroscience, Uppsala University, Uppsala, Sweden; ^2^Institute for Translational Medicine and Biotechnology, Sechenov First Moscow State Medical University, Moscow, Russia

**Keywords:** major depression, L-Dopa, negative geotaxis, CG4269, CG6821, climbing behavior

## Abstract

More than 320 million people live with depression in the world, a disorder that severely limits psychosocial functioning and diminishes quality of life. The prevalence of major depression is almost two times higher in women than in men. However, the molecular mechanisms of its sex-specific pathophysiology are still poorly understood. *Drosophila melanogaster* is an established model for neurobiological research of depression-like states, as well as for the study of molecular and genetic sex differences in the brain. Here, we investigated sex-specific effects on forced-climbing locomotion (negative geotaxis) and gene expression of a fly model of depression-like phenotypes induced by levodopa administration, which was previously shown to impair normal food intake, mating frequency, and serotonin concentration. We observed that both males and females show deficits in the forced-climbing paradigm; however, modulated by distinct gene expression patterns after levodopa administration. Our results suggest that *Drosophila* models can be a valuable tool for identifying the molecular mechanisms underlying the difference of depressive disorder prevalence between men and women.

## Introduction

Depression is a highly impairing disease that affects more than 320 million people worldwide World Health Organization [WHO], 2017. The symptoms of depression can be complex and vary widely between people. However, feelings of hopelessness and anhedonia have been proposed as core manifestations of this disorder (Pettorruso et al., 2020). Based on these features, behavioral despair tests are popular assays for the assessment of animal models of depression phenotypes (Czéh et al., 2016). These assays normally elicit a response of acceptance behavior in the tested organisms, as they endure repeated aversive stimuli beyond their control. Additionally, the fruit fly, *Drosophila melanogaster*, is a consolidated model for the study of neurological disorders (Moulin et al., 2021), including dopaminergic and serotonergic dysfunction in depressive-like states (Kasture et al., 2018). Valuable mechanistic insights were provided by this organism regarding the neurobiology of the observed phenotypes, as general inactivity and anhedonia (Ries et al., 2017). Nevertheless, many other behavioral paradigms are yet unexplored.

Negative geotaxis is an innate escape response, which is elicited when flies are in a confined environment and tapped to the bottom, as they will climb the wall of the container in hope to escape. Previous studies already report automated methods for measuring negative geotaxis in *Drosophila* (Liu et al., 2015; Cao et al., 2017). These approaches rely on video snapshots of vials with multiple flies, providing average climbing distance values in a given period of time. Nevertheless, many applications of this assay would benefit from information on the individual locomotion of the animals, rather than an overall measure of the climbing ability. Thus, by employing commercially available equipment that uses infrared beams for precise position estimation, we developed an automated, high-throughput, protocol that assesses movements after single-fly vial agitation. As this method measures the activity related to the negative geotaxis response while periodically tapping the animals to the bottom of the vial, here we call this feature forced-climbing locomotion.

Pharmacological models for inducing dopaminergic misbalances and depression symptoms in *D. melanogaster* are emerging in the literature. Both the antagonist of dopamine receptors, chlorpromazine, and the dopamine precursor levodopa were shown to induce depression-like phenotypes, such as decreases in food intake and mating frequency (Jiang et al., 2017). Although these drugs have different effects on the dopaminergic system, both can impair serotonergic transmission. Acute levodopa induces a decrease serotonin production by competing with tryptophan for uptake by the amino-acid transporter on 5-HT neurons (Stansley and Yamamoto, 2015), while short-term administration of chlorpromazine has a known antagonist effect on 5-HT2A receptors and can reduce extracellular serotonin levels (Suzuki et al., 2013). Accordingly, exposure to either levodopa or chlorpromazine induces a decrease of 5-HT concentrations in flies (Jiang et al., 2017). Our group recently showed that transient administration of levodopa to young adult flies induces later-life overfeeding behavior, which can be passed to descendants, especially affecting the female offspring (Moulin et al., 2020). However, the extent of the levodopa-induced depression-like behaviors, including differences between males and females, is still not well understood.

Chlorpromazine treatment in flies was shown to significantly change the expression of several genes, which were identified by RNA-seq analysis and confirmed by quantitative RT-PCR (Jiang et al., 2017). The most upregulated gene in this model was CG4269, which had a 20-fold increase in transcription. CG4269, an uncharacterized protein-coding gene, has been shown to be a socially-responsive in *Drosophila*, as male–female interaction downregulates its expression (Ellis and Carney, 2011). Additionally, CG6821 underwent the largest downregulation, with about a 0.01-fold expression level compared to controls. This gene codes for the Lsp1γ protein, an enzyme involved in the storage of amino acids during development (Massey et al., 1997), and shown to be downregulated in a *Drosophila* Parkinson’s disease model (Xun et al., 2008). Interestingly, treatment with lithium chloride, a widespread drug for mood disorders, is able to increase the expression of Lsp1γ (Kasuya et al., 2009). Nevertheless, it is still unclear if these genes are similarly regulated in the levodopa model, which yields similar depression-like behavioral phenotypes, or whether the changes in expression are comparable among sexes.

In this study, we aimed to further characterize the levodopa-induced depression model by implementing an automated version of the *Drosophila* negative geotaxis test, employed to test mobility and climbing motivation in face of inescapable stress. We then examined the transcriptional regulation of CG4269 and CG6821 by performing a quantitative RT-PCR analysis in male and female flies after levodopa administration.

## Methods

### Fly Strains and Maintenance

**FIGURE 1:**
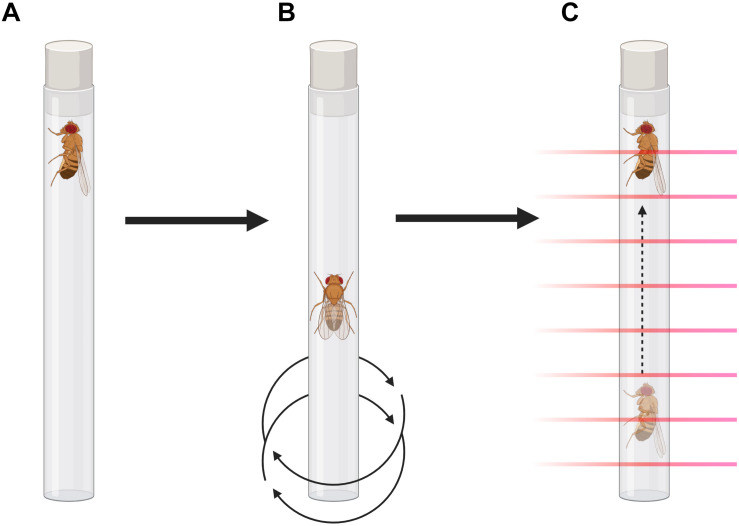
Forced-climbing protocol illustration. **(A)** Flies are placed vertically in individual tubes for measurement in the MB5-Monitor. **(B)** A programmable vortex plate agitates the apparatus for 4 s, each minute, in a 6-h protocol. **(C)** Climbing is assessed by the MB5-Monitor by recording beam-to-beam transitions.

For the experiments, lab wild-type CSORC-strain *D. melanogaster* adults were used, originated from crossing CantonS and OregonR-C flies (Bloomington *Drosophila* Stock Center, Bloomington, IN, United States), kept at 25°C, 12:12-h light/dark, 60% humidity. Flies were fed with Jazz-Mix *Drosophila* food, complemented with 8.3% yeast extract (both from Fisher Scientific, Gothenburg, Sweden). Similar to previous reports (Jiang et al., 2017; Moulin et al., 2020), 5 days post-eclosion male and female flies were collected and transferred to food containing 1 mM levodopa (Sigma-Aldrich, Stockholm, Sweden) for 2 days before the experiments (Figure 1).

### Forced-Climbing Locomotion

Flies were anesthetized with CO_2_ and placed in individual glass tubes covered with cotton on one end and the other end with a black plastic lid. They were loaded vertically into the MB5-Monitor from Trikinetics (Waltham, MA, United States), in which the locomotion of the flies is individually monitored by 17 separate infrared beams for each tube. Each minute, the monitors were vigorously agitated for 4 s by a programmable vortex mounting plate (VMP-MB, Trikinetics), so the flies were tapped to the bottom of the tube, in a protocol that lasted 6 h. To avoid confounders due to circadian rhythmicity, all the experiments were performed during the light phase, from 1 p.m. to 7 p.m. (ZT5 to ZT11). The raw data were converted to CSV files using the DamFileScan software, as described in Pfeiffenberger et al. (2010). Using Microsoft Excel, the number of beam-to-beam transitions (moves) was recorded and averaged for each hour, excluding the 4 s of agitation for each minute. Statistical analyses were performed using GraphPad Prism 8 for Windows (GraphPad Software, San Diego, CA, United States).

### General Activity Assay

For general locomotion measures, after the pharmacological intervention, flies were anesthetized with CO_2_ and put individually in plastic tubes containing regular food without levodopa, which were then placed horizontally in the Drosophila Activity Monitoring System (DAMS, TriKinetics). The tube ends were sealed with regular fly food at one end and a small cotton bud at the other end. Flies were maintained in a 12:12-h light: dark cycle for 3 days, starting at lights on. Next, the raw data were converted to CSV files using the DamFileScan software. For assessing acute changes in locomotion, we manually extracted the mean activity score per hour, for the first 7 h. Then, the Sleep and Circadian Analysis MATLAB Program (SCAMP) by Vecsey Lab was used to analyze the data and evaluate the activity behavior over the course of the 3 days. Statistical analyses were performed using GraphPad Prism 8 for Windows (GraphPad Software, San Diego, CA, United States).

### Quantitative RT-PCR

To evaluate CG4269 and CG6821 transcription, we performed RNA purification, cDNA synthesis, and qRT-PCR, as previously described (Williams et al., 2020). Briefly, the phenol–chloroform method was used for RNA extraction from homogenized tissue samples (25 flies each). RNA concentration was measured using a Nanodrop ND-1000 spectrophotometer (Saveen Werner). cDNA was synthesized using dNTP 20 mM (Fermentas Life Science), random hexamer primers, and M-MLV Reverse Transcriptase (200 U/μl, Invitrogen), following manufacturer instructions. Then, the relative expression levels of a housekeeping gene (RpL32) and the genes of interest were determined by qRT-PCR. Primers were designed based on the Fly Primer Bank^1^. Analysis of qRT-PCR data was performed using MyIQ-1.0 software (Bio-Rad). The following primers (Thermo Fisher Scientific, Germany) were used (forward and reverse sequences): CG6821 (5′-CGGCACCAACACTATCAA-3′, 5′-AAGGCGTTATGCGGC TC-3′); CG6821 (5′-CCCAGCGAATACCACGG-3′, 5′-CGGCACCAGTCCTTGATG-3′); RpL32 (5′-AGCATACA GGCCCAAGATCG-3′, 5′-TGTTGTCGATACCCTTGGGC-3′).

## Results

**FIGURE 2:**
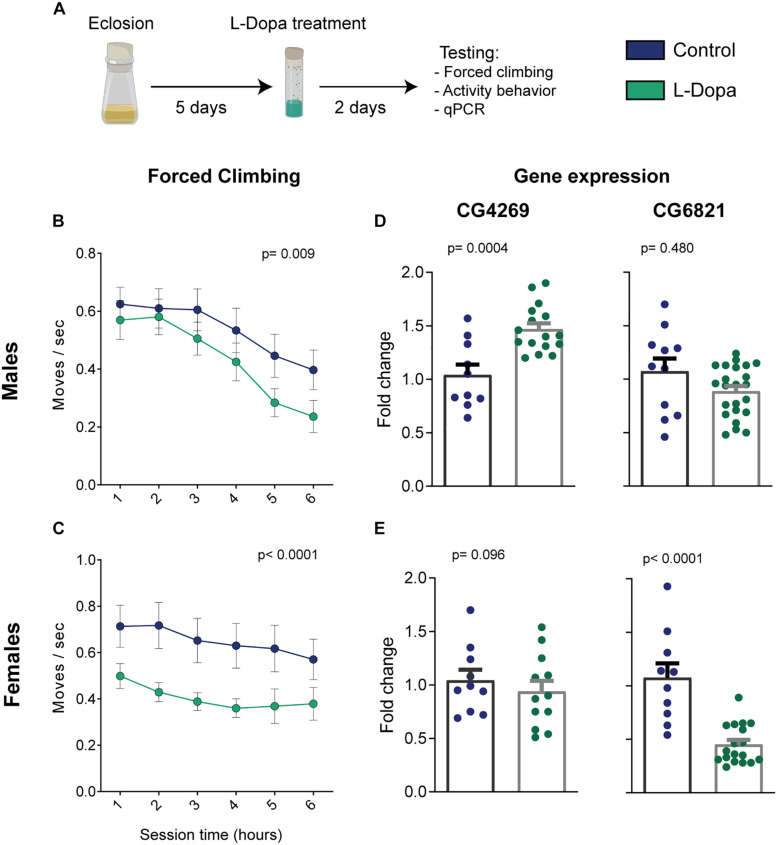
Levodopa-induced phenotypic and genotypic alterations from male and female flies. **(A)** Illustration of the intervention protocol. **(B)** Male and **(C)** female climbing behavior for each hour (*n* = 30/23 and 31/24 control/treated flies, respectively; treatment-effect *p*-values were calculated by two-way ANOVA). **(D,E)** Relative expression of key genes related to the depression model [*n*(CG4269-males) = 10/16; *n*(CG4269-females) = 10/12; *n*(CG6821-males) = 11/23; *n*(CG6821-females) = 10/18 control/treated samples of 25 flies; Student’s *t* test; all groups passed the D’Agostino and Pearson test for normal distribution]. Bars and errors represent mean ± SEM.

For the forced climbing paradigm, we could observe that both male and female control flies reduce the number of moves with time (Figures 2B,C). This may suggest a gradual loss of climbing motivation, either by habituation or by the presence of acceptance-like behavior, as described for popular protocols of unescapable stress (Czéh et al., 2016). Moreover, levodopa administration significantly impaired the climbing response when considering the whole test duration [two-way ANOVA, *p*(male) = 0.009 and *p*(female) < 0.0001 for treatment effects; *p*(male) < 0.0001 and *p*(female) < 0.581 for time effects; *p*(male) = 0.899 and *p*(female) = 0.993 for interaction]. When investigating differences between groups for each individual hour, Holm–Sidak’s *pot hoc* analyses did not yield any statically significant results due to the high variability in the flies’ behavior and multiple-comparison corrections.

Next, we assessed the expression levels of the genes CG4269 and CG6821 by qRT-PCR in a new cohort of flies under the same treatment regime, but that was not tested in the forced climbing assay. As with the dopaminergic antagonist depression model (Jiang et al., 2017), levodopa administration induced overexpression of CG4269 in male flies (Figure 2D left, *p* = 0.0004). However, no significant difference in expression was observed in females (Figure 2E left, *p* = 0.096). Furthermore, CG6821 expression, shown to be reduced in the chlorpromazine model (Jiang et al., 2017), was unchanged for levodopa-treated males (Figure 2D right, *p* = 0.480). Interestingly, female flies exhibited a substantial decrease in CG6821 expression (Figure 2E right, *p* < 0.0001).

**FIGURE 3:**
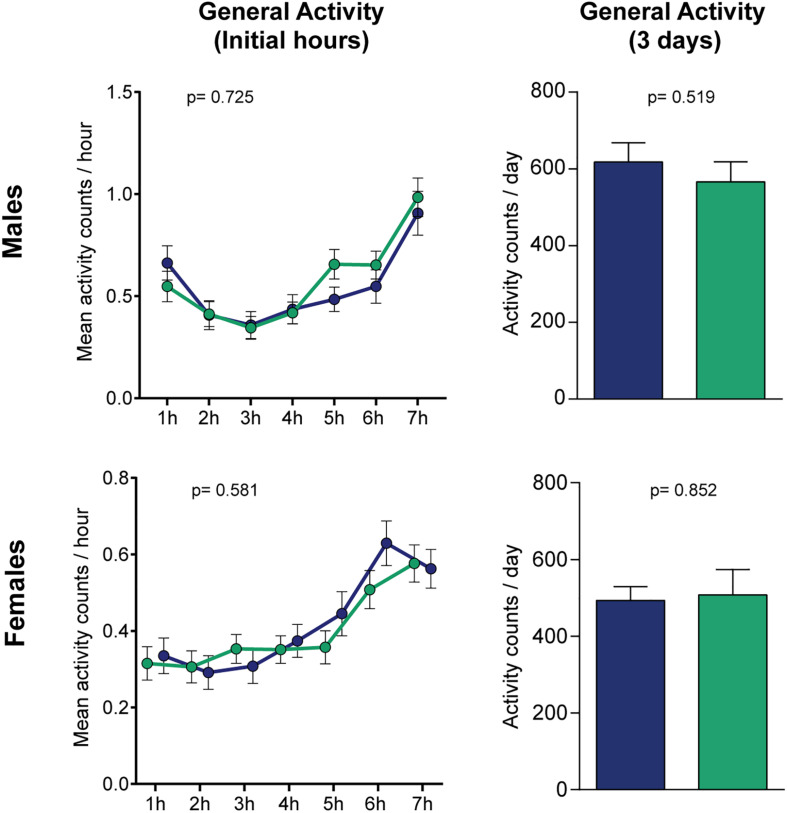
Levodopa does not affect normal locomotion in flies. No statistical differences were found when measuring the activity in horizontal tubes, neither for the initial hours (left panels, treatment-effect *p*-values were calculated by two-way ANOVA) or the mean daily locomotion of the flies when measured for 3 days (right panels, *p*-values calculated by Student’s *t* test). Blue and green bars represent controls and L-Dopa treatment, respectively (*n* = 64/32 and 59/16 control/treated flies for males and females). Bars and errors represent mean ± SEM.

To verify if the levodopa-induced changes in the forced-climbing assay are specific for the negative geotaxis assay, rather than deficits in normal locomotion, we performed a general activity test as shown in a preceding study of our group (Moulin et al., 2020). Levodopa administration did not yield any observable effects in the flies’ activity, neither for the initial hours, where the time frame is comparable to the forced-locomotion assay [two-way ANOVA, *p*(male) = 0.725 and *p*(female) = 0.581 for treatment effects; *p*(male) < 0.0001 and *p*(female) < 0.0001 for time effects; *p*(male) = 0.203 and *p*(female) = 0.222 for interaction], or for the average daily locomotion of the flies when measured for 3 days (Figure 3). These results suggest that the reduced climbing caused by levodopa intake likely reflects a specific impairment in climbing, but not general locomotion.

## Discussion

In this study, we implemented an automated equipment for measuring forced-climbing locomotion in flies. The assay relies on the negative geotaxis of these animals, which is the innate motivation to climb vertically when startled. Using infrared beams for detecting the flies’ movements, we were able to detect impairment in the beam-to-beam transitions, which reflect either slower movements toward the top of the tube, or less activity as the flies disrupt their normal climbing. Impairments in this behavior have been related to lower serotonin and octopamine levels (Meichtry et al., 2020). However, to the best of our knowledge, it is the first time this test is used in an automated way to assess a *Drosophila* model of depression.

We observed that administration of levodopa for 48 h impaired the forced-climbing locomotion in adult flies of both sexes. These results are relatable to what is observed in rodents, where levodopa intake causes increased forced-swimming immobility (Borah and Mohanakumar, 2007). On the other hand, general activity is unaffected by the same treatment, which differs from previous stress-induced fly models of depression (Ries et al., 2017). These results suggest that levodopa does not induce severely reduced physical endurance, which could influence the tapping-induced negative geotaxis behavior. However, our assessment is not able to indicate whether or not this impairment is due to fine motor damage or reduced escape motivation. An alternative explanation also not covered by our experiments is that levodopa treatment might alter the startle stimuli’ perception or differentially affect the subsequent locomotion. Thus, we cannot rule out an effect on the fly startle response or the endurance of flies in a climbing assay. Yet, these findings expand the list of numerous phenotypes previously published on this pharmacological model, which can be further explored in future studies.

Interestingly, there were noteworthy behavioral differences between sexes during the forced-climbing assay. First, both groups of male flies have comparable levels of locomotion at the initial hours of the assay, while a clearer treatment effect is observed at the final hours. Female flies treated with levodopa, on the other hand, displayed impaired climbing already in the first hour of the assay. These differences may be a consequence of divergent amounts of food consumption or drug sensitivity, as it was demonstrated before that levodopa had a bigger effect on reducing food intake in males than females (Jiang et al., 2017). Additionally, we can observe distinct rates of decrease in climbing activity with time, as there was a statistically significant impact of time for males, but not females. This lack of statistical significance may come from a “floor effect,” as treated flies show significantly reduced activity levels at the initial hours, with a slight further decrease afterward. Nevertheless, the possibility that female flies are more resistant to the current automated climbing assay should be considered.

It is possible that the reduction in climbing activity observed in control males and females is originated from learned helplessness, where uncontrollable stress causes downregulation of escape responses. This phenomenon is linked to depression-like behavior in animals, and has been already described in flies (Yang et al., 2013). However, some limitations to this interpretation should be considered. First, it is not clear if the negative geotaxis behavior after tapping, measured in our assay, is equivalent to the escape response to aversive stimuli assessed in learned helplessness tests. Second, our assay did not test if the flies remained unmotivated to climb in the absence of the stressful events (i.e., after the tapping is interrupted), which is a central paradigm of learned helplessness. Further characterization is needed to assert if consecutive tapping and subsequent reduction of negative geotaxis correspond to a depression-like state.

Levodopa is shown to induce aberrant connectivity of 5-HT neurons and to reduce serotonin levels in the *Drosophila* brain (Niens et al., 2017), which can be the origin of the observed phenotype. Despite undergoing the same intervention, our findings suggest that sex-specific genetic alterations occur in flies, which nevertheless develop similar behavioral impairments. These results should be interpreted keeping in mind that expression changes of CG4269 and CG6821 in male flies were previously observed after administration of a higher dose of the dopaminergic receptors’ antagonist chlorpromazine (2,000 mg/L–5.6 mM, Jiang et al., 2017). Thus, although the effects of these drugs cannot be directly compared, the differential gene expression observed for the levodopa treatment may be due to sex-specific sensitivity to the drug, posing the lack of a dose–response curve as a limitation of our data. Moreover, as these tests were performed separately, the effects of levodopa on gene expression should not be interpreted as the cause of the observed climbing behavior deficits. Nevertheless, to the best of our knowledge, these are the first results showing that gene expression can be divergent among males and females from a *D. melanogaster* depression model.

Accordingly, it is accepted that many psychiatric disorders are multifactorial, as several different pathways could lead to similar symptoms (Pavão et al., 2015; Pinto et al., 2017). Thus, understanding the sex-specific molecular underpinnings of these conditions is imperative in order to develop more effective and personalized interventions. Our results suggest that the *Drosophila* may provide useful insights when assessing the genotypic sex differences in preclinical models of depression-like phenotypes. Nevertheless, future research is warranted to investigate if conserved pathways or orthologs are likewise affected and further determine the model translational value.

## Data Availability Statement

The original contributions presented in the study are included in the article/Supplementary Material, further inquiries can be directed to the corresponding author/s.

## Author Contributions

TM, MW, and HS: conceptualization and manuscript writing. TM: methodology. TM, FF, PC, and AH: formal analysis and investigation. MW and HS: supervision and funding acquisition. TM and MW: project administration. All authors read and agreed to the published version of the manuscript.

## Conflict of Interest

The authors declare that the research was conducted in the absence of any commercial or financial relationships that could be construed as a potential conflict of interest.

## Footnotes

1 www.flyrnai.org/flyprimerbank
